# Toward Using Twitter for PrEP-Related Interventions: An Automated Natural Language Processing Pipeline for Identifying Gay or Bisexual Men in the United States

**DOI:** 10.2196/32405

**Published:** 2022-04-25

**Authors:** Ari Z Klein, Steven Meanley, Karen O'Connor, José A Bauermeister, Graciela Gonzalez-Hernandez

**Affiliations:** 1 Department of Biostatistics, Epidemiology, and Informatics Perelman School of Medicine University of Pennsylvania Philadelphia, PA United States; 2 Department of Family and Community Health School of Nursing University of Pennsylvania Philadelphia, PA United States

**Keywords:** natural language processing, social media, data mining, PrEP, pre-exposure prophylaxis, HIV, AIDS

## Abstract

**Background:**

Pre-exposure prophylaxis (PrEP) is highly effective at preventing the acquisition of HIV. There is a substantial gap, however, between the number of people in the United States who have indications for PrEP and the number of them who are prescribed PrEP. Although Twitter content has been analyzed as a source of PrEP-related data (eg, barriers), methods have not been developed to enable the use of Twitter as a platform for implementing PrEP-related interventions.

**Objective:**

Men who have sex with men (MSM) are the population most affected by HIV in the United States. Therefore, the objectives of this study were to (1) develop an automated natural language processing (NLP) pipeline for identifying men in the United States who have reported on Twitter that they are gay, bisexual, or MSM and (2) assess the extent to which they demographically represent MSM in the United States with new HIV diagnoses.

**Methods:**

Between September 2020 and January 2021, we used the Twitter Streaming Application Programming Interface (API) to collect more than 3 million tweets containing keywords that men may include in posts reporting that they are gay, bisexual, or MSM. We deployed handwritten, high-precision regular expressions—designed to filter out noise and identify actual self-reports—on the tweets and their user profile metadata. We identified 10,043 unique users geolocated in the United States and drew upon a validated NLP tool to automatically identify their ages.

**Results:**

By manually distinguishing true- and false-positive self-reports in the tweets or profiles of 1000 (10%) of the 10,043 users identified by our automated pipeline, we established that our pipeline has a precision of 0.85. Among the 8756 users for which a US state–level geolocation was detected, 5096 (58.2%) were in the 10 states with the highest numbers of new HIV diagnoses. Among the 6240 users for which a county-level geolocation was detected, 4252 (68.1%) were in counties or states considered priority jurisdictions by the *Ending the HIV Epidemic* initiative. Furthermore, the age distribution of the users reflected that of MSM in the United States with new HIV diagnoses.

**Conclusions:**

Our automated NLP pipeline can be used to identify MSM in the United States who may be at risk of acquiring HIV, laying the groundwork for using Twitter on a large scale to directly target PrEP-related interventions at this population.

## Introduction

Pre-exposure prophylaxis (PrEP) with antiretroviral drugs is highly effective at preventing the acquisition of HIV in men who have sex with men (MSM) [1]. There is a substantial gap, however, between the number of people in the United States who have indications for PrEP, including 25% of MSM [2], and the number of them who are prescribed PrEP [3]; approximately one-third of primary care physicians (PCPs) in the United States who are aware of PrEP have prescribed PrEP or referred a patient for PrEP [4]. Although efforts should be made to increase PCPs’ adoption of PrEP recommendations into routine clinical practice, PCP-based interventions are limited because some MSM, especially younger men, face challenges when disclosing their same-sex sexual behaviors to their PCPs [5]. Based on the findings of a recent study by Reuter et al [6] that examined Twitter users’ attitudes toward being monitored for health-related research, some MSM may be more open to PrEP-related interventions on social media, such as targeted messages or advertisements.

Hannaford et al [7] found that social media can help identify factors for implementing PrEP-related interventions that are not captured by traditional research methods, and they suggested that social media may present novel opportunities to implement PrEP-related interventions. Although Twitter content has been analyzed as a source of PrEP-related data (eg, barriers) [8,9], to our knowledge, methods have not been developed to enable the use of Twitter as a platform for PrEP-related interventions. The foremost requirement for implementing PrEP-related interventions on Twitter is to identify users in the populations that have indications for PrEP. Given that MSM are the population most affected by HIV in the United States [10], the objectives of this study were to (1) develop an automated natural language processing (NLP) pipeline for identifying men in the United States who have reported on Twitter that they are gay, bisexual, or MSM and (2) assess the extent to which they demographically represent MSM in the United States with new HIV diagnoses. This study seeks to lay the groundwork for using Twitter on a large scale to directly target PrEP-related interventions at MSM who may be at risk of acquiring HIV.

## Methods

### Ethical Considerations

The Institutional Review Board of the University of Pennsylvania reviewed this study and deemed it exempt human subjects research under Category (4) of Paragraph (b) of the US Code of Federal Regulations Title 45 Section 46.101 for publicly available data sources (45 CFR §46.101(b)(4)).

### Data Collection

Between September 2020 and January 2021, we used the Twitter Streaming Application Programming Interface (API) to collect more than 3 million tweets containing keywords that men may include in posts reporting that they are gay, bisexual, or MSM. As a preliminary approach, we deployed handwritten, high-precision regular expressions—search patterns designed to automatically match text strings—on the 3 million tweets to filter out noise and identify actual self-reports (Multimedia Appendix 1). After automatically removing retweets and “reported speech” (eg, quotations, news headlines) [11], the regular expressions matched 8603 tweets that were posted by 6358 users geolocated in the United States [12].

**Figure 1:**
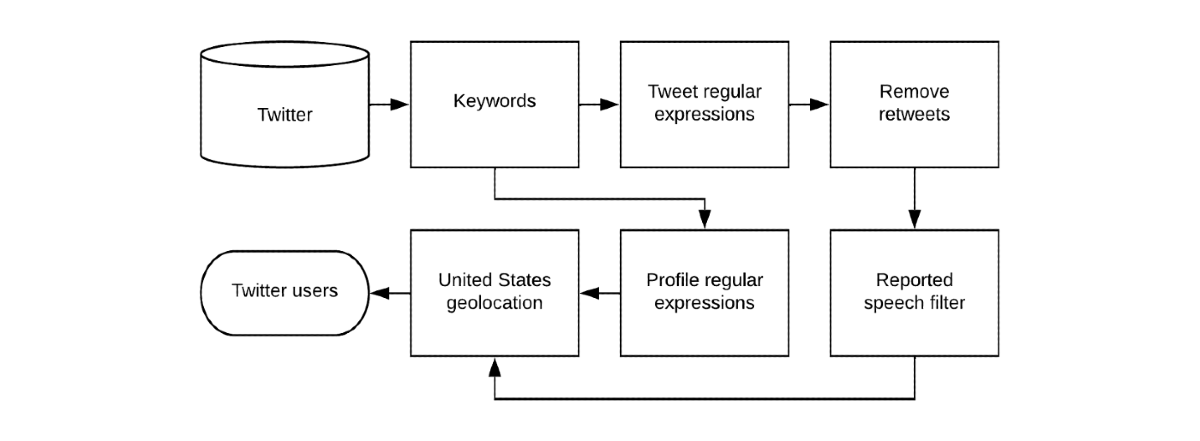
Automated natural language processing pipeline for identifying men in the United States who have reported on Twitter that they are gay, bisexual, or men who have sex with men.

In addition to tweet-based regular expressions, we also deployed handwritten regular expressions on the user profile metadata of the 3 million tweets collected from the Twitter Streaming API (Multimedia Appendix 1). The regular expressions matched the profile metadata of 4127 users geolocated in the United States [12]. After removing duplicate users from our tweet- and profile-based searches, we identified a total of 10,043 unique users. Figure 1 illustrates our automated pipeline for identifying men in the United States who have reported on Twitter that they are gay, bisexual, or MSM. To assess the extent to which they demographically represent MSM in the United States with new HIV diagnoses, we analyzed the state- and county-level geolocations [12] of these 10,043 users and drew upon a validated NLP tool [13] to automatically identify their ages.

## Results

### Pipeline Evaluation

True positives and false positives were manually distinguished by 2 annotators in a random sample of 1000 (10%) of the 10,043 users that were identified by our automated pipeline, consisting of 500 matching tweets and 500 matching profiles. *True positives* were defined as tweets or profiles in which the users reported that they are gay, bisexual, or MSM. Overall interannotator agreement (Cohen κ) based on independent, dual annotations for all 1000 users was 0.81, which is deemed to be “almost perfect agreement” [14]. More specifically, interannotator agreement was 0.83 for the 500 tweets and 0.79 for the 500 profiles. Upon resolving the disagreements, 417 (83.4%) tweets and 430 (86%) profiles were annotated as true positives and 83 (16.6%) tweets and 70 (14%) profiles were annotated as false positives. Based on this evaluation, our automated pipeline has an overall precision of 0.85, where *precision = true positives / (true positives + false positives)*. Table 1 provides examples of tweets and profiles that were manually annotated as true or false positives. The majority of the profiles that were annotated as false positives were users that mentioned being transgender or nonbinary—populations that are beyond the scope of this study.

**Table 1:** Sample manual annotations of tweets and profiles.

Type	Text	Label
Tweet	End the FDA’s discriminatory and unscientific policy against gay men like me donating blood.	True positive
Tweet	As a bi guy we get so little representation, and almost all of its negative. It’s frustrating.	True positive
Tweet	Today, we remember Matthew Shepard who’s life was cut short as a result of a hate crime due to his identity as a gay male.	False positive
Profile	A proud black gay guy.	True positive
Profile	50+ gay trans man, writer, film and food lover. He/him OR they/them.	False positive

### Demographics

**Figure 2:**
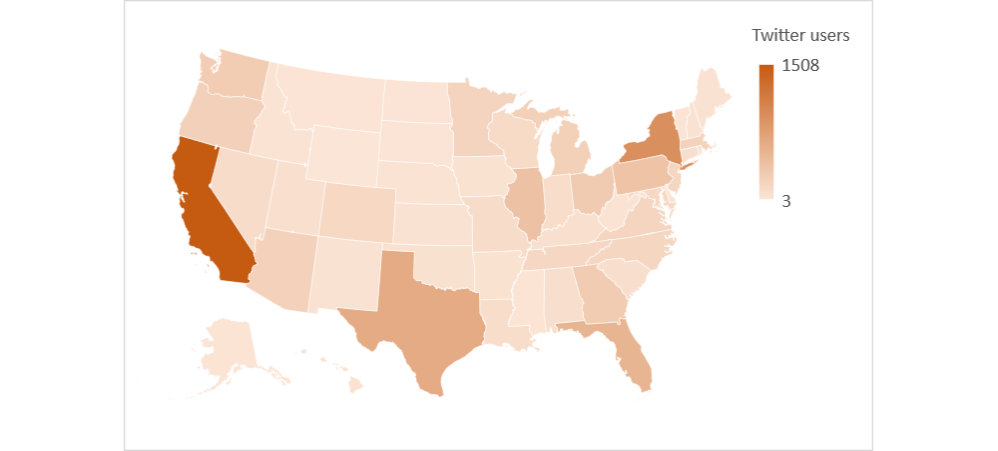
Number of Twitter users, by state, identified by our automated pipeline between September 2020 and January 2021.

To assess the utility of our automated pipeline for identifying MSM in the United States who may be particularly at risk of acquiring HIV, we analyzed their state- and county-level geolocations and ages. We detected a US state–level geolocation for 8756 (87.6%) of the 10,043 users identified by our automated pipeline, including users from all 50 states and the District of Columbia. As Figure 2 illustrates, the largest numbers of users were detected in California, New York, Texas, Florida, Illinois, Pennsylvania, Ohio, and Georgia. We detected a county-level geolocation for 6240 (71.2%) of these 8756 users. Table 2 presents the 15 counties for which we detected at least 100 users. We detected an age of ≥13 years [10] for 4782 (47.6%) of the 10,043 users, with a mean age of 31.9 (SD 13.1) years and a median age of 29 years. Table 3 presents the age distribution, based on each user’s most recent tweet containing a self-report of age.

**Table 2:** Counties with at least 100 Twitter users identified by our automated pipeline between September 2020 and January 2021.

US county	Users (N=6240), n (%)
Los Angeles County, CA	535 (8.6)
New York County, NY	417 (6.7)
Cook County, IL	318 (5.1)
District of Columbia, DC	237 (3.8)
King County, WA	192 (3.1)
Fulton County, GA	155 (2.5)
San Mateo County, CA	151 (2.4)
Multnomah County, OR	128 (2.1)
Kings County, NY	127 (2)
Dallas County, TX	123 (2)
Philadelphia County, PA	121 (1.9)
Harris County, TX	116 (1.9)
Maricopa County, AZ	111 (1.8)
Suffolk County, MA	110 (1.8)
Travis County, TX	109 (1.7)

**Table 3:** Age distribution of Twitter users identified by our automated pipeline between September 2020 and January 2021.

Age group (years)	Users (N=4782), n (%)
13-24	1630 (34.1)
25-34	1644 (34.4)
35-44	704 (14.7)
45-54	449 (9.4)
≥55	355 (7.4)

## Discussion

### Principal Findings

Our study demonstrates that gay men, bisexual men, or MSM in the United States publicly report their sexual orientation on Twitter and that these users can be accurately identified on a large scale. Moreover, among the 8756 users for which our automated pipeline detected a US state–level geolocation, 5096 (58.2%) were in the 10 states with the highest numbers of new HIV diagnoses [10]. Among the 6240 users for which a county-level geolocation was detected, 4252 (68.1%) were in counties or states considered priority jurisdictions by the *Ending the HIV Epidemic* initiative [15]. Furthermore, the age distribution of the users reflected the ranking of the most frequent age groups with new HIV diagnoses among MSM in the United States [10], with the 25-34 years age group first and the 13-24 years age group second. More specifically, these 2 age groups represent both the majority of the users in this study and the majority of MSM with new HIV diagnoses [10]. The mean (31.9 years) and median (29 years) ages of the users are within the age group (25-34 years) with the largest number of new HIV diagnoses, which is also the only age group in which HIV infections have increased since 2014 [10]. Therefore, our automated pipeline can be used as the basis for PrEP-related interventions targeted directly at MSM who are largely in the regions and age groups most affected by HIV in the United States, including younger men who may face challenges when discussing their same-sex sexual behaviors with their PCPs [5].

### Conclusions

This paper presented an automated NLP pipeline that can be used to identify MSM in the United States who may be at risk of acquiring HIV, laying the groundwork for using Twitter on a large scale to directly target PrEP-related interventions at this population.

## Appendix

Multimedia Appendix 1 Regular expressions.

## Abbreviations

API: application programming interface
MSM: men who have sex with men
NLP: natural language processing
PCP: primary care physician
PrEP: pre-exposure prophylaxis

## References

[1] Grant RM, Lama JR, Anderson PL, McMahan V, Liu AY, Vargas L, Goicochea P, Casapía Martín, Guanira-Carranza JV, Ramirez-Cardich ME, Montoya-Herrera O, Fernández Telmo, Veloso VG, Buchbinder SP, Chariyalertsak S, Schechter M, Bekker L, Mayer KH, Kallás Esper Georges, Amico KR, Mulligan K, Bushman LR, Hance RJ, Ganoza C, Defechereux P, Postle B, Wang F, McConnell JJ, Zheng J, Lee J, Rooney JF, Jaffe HS, Martinez AI, Burns DN, Glidden DV, iPrEx Study Team (2010). Preexposure chemoprophylaxis for HIV prevention in men who have sex with men. N Engl J Med.

[2] Smith DK, Van Handel M, Wolitski RJ, Stryker JE, Hall HI, Prejean J, Koenig LJ, Valleroy LA (2015). Vital signs: estimated percentages and numbers of adults with indications for preexposure prophylaxis to prevent HIV acquisition--United States, 2015. MMWR Morb Mortal Wkly Rep.

[3] Huang YA, Zhu W, Smith DK, Harris N, Hoover KW (2018). HIV preexposure prophylaxis, by race and ethnicity - United States, 2014-2016. MMWR Morb Mortal Wkly Rep.

[4] Blackstock OJ, Moore BA, Berkenblit GV, Calabrese SK, Cunningham CO, Fiellin DA, Patel VV, Phillips KA, Tetrault JM, Shah M, Edelman EJ (2017). A cross-sectional online survey of HIV pre-exposure prophylaxis adoption among primary care physicians. J Gen Intern Med.

[5] Petroll AE, Mitchell JW (2015). Health insurance and disclosure of same-sex sexual behaviors among gay and bisexual men in same-sex relationships. LGBT Health.

[6] Reuter K, Zhu Y, Angyan P, Le N, Merchant AA, Zimmer M (2019). Public concern About monitoring Twitter users and their conversations to recruit for clinical trials: survey study. J Med Internet Res.

[7] Hannaford A, Lipshie-Williams M, Starrels JL, Arnsten JH, Rizzuto J, Cohen P, Jacobs D, Patel VV (2018). The use of online posts to identify barriers to and facilitators of HIV pre-exposure prophylaxis (PrEP) among men who have sex with men: a comparison to a systematic review of the peer-reviewed literature. AIDS Behav.

[8] McLaughlin ML, Hou J, Meng J, Hu CW, An Z, Park M, Nam Y (2016). Propagation of information about preexposure prophylaxis (PrEP) for HIV prevention through Twitter. Health Commun.

[9] Schwartz J, Grimm J (2017). PrEP on Twitter: information, barriers, and stigma. Health Commun.

[10] Centers for Disease Control and Prevention (2020). Diagnoses of HIV infection in the United States and dependent areas, 2018 (Updated). HIV Surveillance Report, 2018 (Updated).

[11] Klein AZ, Cai H, Weissenbacher D, Levine LD, Gonzalez-Hernandez G (2020). A natural language processing pipeline to advance the use of Twitter data for digital epidemiology of adverse pregnancy outcomes. J Biomed Inform.

[12] Dredze M, Paul MJ, Bergsma S, Tran H (2013). Carmen: a Twitter geo-location system with applications to public health.

[13] Klein AZ, Magge A, Gonzalez-Hernandez G (2022). ReportAGE: Automatically extracting the exact age of Twitter users based on self-reports in tweets. PLoS One.

[14] Viera AJ, Garrett JM (2005). Understanding interobserver agreement: the kappa statistic. Fam Med.

[15] Centers for Disease Control and Prevention. Ending the HIV epidemic in the U.S. - Jurisdictions.

