# Supplementary material for: Toward Using Twitter for PrEP-Related Interventions: An Automated Natural Language Processing Pipeline for Identifying Gay or Bisexual Men in the United States
Source: JMIR Public Health Surveill. 2022 Apr 25;8(4):e32405. doi: 10.2196/32405 (PMC9086871; doi:10.2196/32405)
Supplement: Multimedia Appendix 1 [file publichealth_v8i4e32405_app1.docx]

**Regular Expressions**

REGEXES_TWEETS = [

r'\b(dm|send|me|my|i|i\W?m)\b.*#(truvadawhore|gaytop|gayvers|teambottom|teamvers|bareback|teamtop|gaybottom)\b',

r'\b(i\W?m|i\s+am|as)\s+(?!not)(\S+\s+)?a\s+(queer|bi\W?sexual|bi|gay)\s+(man|guy|male|dude|boy)\b',

r'\bmy\s+fellow\s+(queer|bi\W?sexual|bi|gay)\s+(men|guys|males|dudes|boys)\b',

r'\b(queer|bi\W?sexual|bi|gay)\s+(man|men|guy|guys|male|males|dude|dudes|boy|boys)\W+like\s+(me|myself)\b'

]

REGEXES_PROFILE = [

r'\b(queer|bi\W?sexual|bi|gay)\b.*\b(man|guy|male|dude|boy|he|him|his|husband|father|dad)\b',

r'\b(man|guy|male|dude|boy|he|him|his|husband|father|dad)\b.*\b(queer|bi\W?sexual|bi|gay)\b',

r'#(truvadawhore|gaytop|gayvers|teambottom|teamvers|bareback|teamtop|gaybottom)\b'

]
